# Commentary: Catalysts of violence against women students: the role of the university, aggressors, and victims

**DOI:** 10.3389/fpsyg.2025.1595955

**Published:** 2025-08-19

**Authors:** Dulce María Díaz-Sosa, Centli Guillén-Díaz-Barriga, Ana Celia Chapa-Romero, Lorena Alejandra Flores-Plata, Anabel De la Rosa-Gómez

**Affiliations:** ^1^Department of Health Sciences, Metropolitan Autonomous University Campus Lerma (UAM-L), Lerma de Villada, Mexico; ^2^Faculty of Psychology, National Autonomous University of Mexico, Mexico City, Mexico; ^3^Iztacala College of Higher Education, National Autonomous University of Mexico, Mexico City, Mexico

**Keywords:** university context, violence against women, women vulnerability, women strength, victims and aggressors, gender equality, quality education

## Introduction

This work is based on the article by (Barbosa et al. 2024), which highlights the need to deeply address, both theoretically and empirically, the various levels of violence against women. These include the institutional level, the individual level, and the victim level, where the dichotomy between vulnerability and resilience is examined.

The article emphasizes the importance of delving into this last aspect, as well as the role of youth education as a key tool in eradicating gender-based violence. Therefore, the objective of this analysis is to contribute to the reflection on these catalysts and highlight the need to develop effective strategies to combat gender-based violence in educational institutions.

A first point that we wish to explore in this commentary is about Resilience and vulnerability as antecedent factors of violence, in the article it is highlights that women's resilience, understood in terms of economic, educational, and emotional autonomy, can trigger violent reactions from aggressors.

This dynamic has been widely documented by (Faludi 2006), who defines *backlash* as a hostile reaction to the progress achieved by women in both the public and private spheres. This phenomenon manifests through strategies ranging from denying the need for structural changes to justifying violence through narratives that blame victims or minimize gender-based violence (Braithwaite, 2013; Restrepo et al., 2022). Given this scenario, it is imperative to design strategies that counteract the effects of backlash. In this regard, strengthening support networks among women and participating in feminist collectives are key mechanisms for resisting these reactions (Viswanathan, 2021; Cerva Cerna, 2020).

Additionally, it is essential to develop educational arguments that make visible the hidden forms of discrimination and setbacks in terms of rights. It is also crucial to discern which confrontations are strategic, avoiding unnecessary exhaustion in fruitless debates and focusing efforts on educational actions, such as workshops and conferences, that promote democratic values and tools for social change (VicHealth, 2018).

Despite preventive efforts, it is undeniable that a significant percentage of women have experienced violence, leading to severe consequences for their mental health, including the development of Post-Traumatic Stress Disorder (Karataş et al., 2020).

At this point, university education stands out as a key strategy for preventing gender-based violence (Vázquez et al., 2021), making it necessary to thoroughly examine the most effective methodologies in this context.

These changes could be accompanied by critical and ethical pedagogies that promote self-awareness through feeling, thinking, and acting; that strengthen debate and stimulate critical imagination, new ways of relating, and caring for each other in classrooms and other spaces within and outside the university (Chapa Romero et al., 2022).

(McNaughton Reyes et al. 2021), conducted a systematic review of dating violence prevention programs, a public health and human rights issue. Their findings emphasized the need to expand these programs to low-resource contexts. These programs should include education on healthy relationships, gender equity, conflict resolution, and access to community violence support services.

In this context, higher education institutions play a pivotal role in addressing gender-based violence—not only by implementing regulations, but also by fostering an organizational culture that actively promotes equity and ensures comprehensive support for victims.

It is imperative for universities to establish specialized psychological intervention protocols to assist students who are victims of sexual violence, considering the impact of Post-Traumatic Stress Disorder on their emotional wellbeing and academic performance (Gamboa, 2019).

These protocols should be based on scientific evidence and a gender-sensitive approach that includes psychoeducation on power hierarchies, gender roles, and re-victimization processes. In this regard, Trauma-Focused Cognitive Behavioral Therapy has demonstrated significant effectiveness in treating PTSD (Cohen and Mannarino, 1988). It is also recommended to design strategies that ensure women's access to these treatments, as financial and time constraints often hinder adherence. In this context, telepsychology emerges as a viable alternative to expand coverage and accessibility to psychological care (Domínguez-Rodríguez and De la Rosa-Gómez, 2021).

Finally, it is important to involve the community in the design, development, and implementation of effective strategies. In this regard, research has documented the role of organizations composed of university women from various sectors (students, academics, and workers) in driving necessary changes to prevent, address, investigate, sanction, repair, and eradicate gender-based violence in universities (Cerva Cerna, 2020; Chapa Romero et al., 2022).

## Discussion

To effectively address gender-based violence in university settings, it is essential to consider not only individual cases but also the institutional and social responses that perpetuate such violence. Therefore, it is necessary to approach this issue from a gender perspective that allows for the analysis of power relations, normative discourses, and institutional practices that sustain it.

An important point to consider within this expanded framework is the role of violence perpetrated by women in academic environments. Psychological harassment, social exclusion, and the reinforcement of patriarchal norms by female peers or authority figures are forms of violence that often remain invisible. It is crucial to underscore that these dynamics are not separate from patriarchal structural violence; in many cases, they are actually reinforced by it. For example, the high level of competitiveness among women—arising from contexts in which they must work twice as hard as men to receive recognition (Correa et al., 2025)—along with the low representation of women among faculty and students, and the persistence of hegemonic masculinities, create conditions that facilitate such forms of aggression (Barbosa et al., 2024). These types of violence hinder the empowerment and wellbeing of other women. Recognizing their existence is essential for developing more comprehensive prevention strategies and building truly inclusive and equitable academic environments.

Another critical aspect to address is the role of bystanders—students, faculty, and administrative staff—who, despite witnessing or being aware of incidents of gender-based violence, often remain passive or unsure of how to intervene. Such inaction weakens victim protection and contributes to the normalization of violence. The implementation of *bystander intervention* programs, in both in-person and online formats, has been shown to significantly improve self-efficacy, willingness, and actual intervention behaviors among students and faculty (Jouriles et al., 2016; Kettrey and Marx, 2019; Coker et al., 2019), thereby contributing to a reduction in gender-based violence. Furthermore, training sessions targeted at faculty and staff have strengthened institutional awareness, vocabulary on the subject, and intervention capabilities (McMahon et al., 2021). Incorporating this approach is key to fostering a culture of shared responsibility and active prevention within educational institutions.

In conclusion, eradicating gender-based violence in higher education requires visibility of its multiple forms and actors, as well as the development of mechanisms that ensure non-repetition, access to justice, and the reconstruction of both individual and collective life projects for those who have experienced violence (Chapa Romero et al., 2022).
